# Role of big data and technological advancements in monitoring and development of smart cities

**DOI:** 10.1016/j.heliyon.2024.e34821

**Published:** 2024-07-18

**Authors:** Xiyin Ma, Jian Li, Zhiming Guo, Zhonglu Wan

**Affiliations:** a Shanghai Technical Institute of Electronics & Information, Shanghai, 201400, China; b Shanghai Institute of Technology, Shanghai, 201400, China; c Shendu Design Group Co. Ltd., Shanghai, 200092, China; d Jilin Institute of Economics and Finance, China

**Keywords:** Smart cities, Big data analysis, Sustainable development

## Abstract

Driven by rapid advancements in technology and data science, a revolutionary transformation is sweeping across environmentally friendly cities worldwide. This surge stems from a pressing need to tackle the intricate complexities of urban sustainability, encompassing everything from infrastructure and governance to fragmented design and technological solutions. To effectively manage these complexities and accurately measure, assess, and optimize their sustainability performance, sustainable communities are increasingly tapping into the potential of smart city technologies, particularly big data and its fictionalized applications. This trend culminates in the emergence of smart cities. This article delves into the current state of research surrounding data-driven, environmentally conscious smart cities, aiming to assess the extent to which these two concepts are currently being integrated and identify potential gaps in this field. Through a strong emphasis on evidence-based research, the study underscores the potential of big data technologies to offer innovative approaches for monitoring, comprehending, evaluating, and ultimately managing sustainable urban development. It further highlights the crucial role of data-driven advancements in formulating strategic development policies and operational management procedures, ensuring that environmentally conscious cities can continue to contribute to sustainability goals even amidst rapid urbanization.

## 1 Introduction

We are currently living in a technological age where most of our daily activities depend on modern technology and computing devices. These technologies have increasingly become indispensable across various fields, including socioeconomics, natural sciences, sustainability, and climate studies [1]. Recent technological advancements provide robust evidence supporting this claim. For instance, in socioeconomics, cutting-edge data analytics and machine learning algorithms have enabled more precise predictions and analyses of economic trends and market behaviors. In the realm of natural sciences, advanced imaging technologies and computational modeling have transformed our comprehension of intricate systems, such as molecular structures and ecological interactions. Similarly, in sustainability and climate studies, state-of-the-art tools such as remote sensing, big data analytics, and climate models have facilitated thorough assessments of environmental changes, guiding policymaking efforts effectively [2,3]. They aim to enhance the efficiency and productivity of individual systems.

Digital technology is rapidly weaving itself into the fabric of our everyday lives, fueled by the transformation of knowledge into a computer-readable format – a process known as digitization. This meticulous preservation of accumulated wisdom, encompassing historical artifacts, art, and film, has borne abundant fruit under the nurturing hand of information technology. The benefits reaped from digitization have further blossomed into a plethora of interconnected devices and tools, seamlessly integrated into the vast ecosystem of the Internet of Things (IoT). The IoT, a robust network of physical objects bound together by the internet through embedded sensors, software, and diverse technologies, facilitates the seamless transmission and collection of data. This confluence of concurrently developed technologies, including real-time analysis, predictive modeling, and artificial intelligence, allows us to grapple with the burgeoning volumes of information – often referred to as big data. While the full potential of this colossal digital deluge remains untapped, it unlocks extraordinary possibilities for propelling smart, interconnected cities towards greater efficiency and sustainability [4,5].

Digital technology unleashes a powerful arsenal of tools for tackling sustainability challenges. However, navigating this new frontier demands a delicate balancing act between technological advancements and their environmental and social implications. While the potential for intelligent resource management and equitable access to amenities is undeniable [6], several critical hurdles must be addressed to ensure a truly sustainable and equitable future. One major concern lies in the very process of technological creation. Production and development of electronic devices often consume scarce resources and generate mountains of e-waste – unwanted electronics discarded or rarely recycled [7,8]. This highlights the urgent need for sustainable life-cycle assessments and the development of technologies for efficient e-waste recycling. Another potential pitfall lies in the digital divide. Expanding networks and access to the internet, while crucial for bridging development gaps and promoting opportunities like online learning [9], can conversely exacerbate inequalities if equitable infrastructure and connectivity are not prioritized. Unbridled technological advancements risk leaving marginalized communities further behind. Furthermore, the vast collection and transparency of big data, while promising for insightful analytics and decision-making, raises serious concerns about information security and privacy [10,11]. Data breaches and misuse can pose significant risks to individuals and societal trust in new technologies. Addressing these concerns alongside fostering responsible data governance is crucial.

While these challenges cannot be ignored, they should not be viewed as insurmountable roadblocks. Embracing a responsible and holistic approach to digital sustainability offers immense potential. Here are some avenues for navigating these challenges: Implementing closed-loop production systems, promoting responsible manufacturing practices, and investing in e-waste recycling infrastructure can significantly reduce the environmental footprint of digital technologies. Prioritizing equitable access to internet connectivity and digital literacy programs can bridge the digital divide and ensure all communities benefit from the opportunity's technology offers. Establishing robust data protection frameworks, promoting transparency, and investing in cybersecurity measures can mitigate the risks associated with big data while unlocking its potential for sustainable solutions [12,13].

In 2015, the General Assembly of the United Nations (UN) established a strategy for achieving equitable and environmentally friendly development by 2030 [14,15]. The Sustainable Development Goals (SDGs) comprise a list of 17 challenges that need to be overcome in order to achieve the ambitious international objective of a better world. These goals include eradicating poverty and hunger, ensuring good health and well-being for all, providing access to quality education, promoting sustainable cities and communities, adopting ethical production and consumption practices, acting on climate change, preserving life below water and on land, fostering stability, equity, and resilient institutions, and promoting partnerships for the goals. Goal 6 focuses on ensuring access to clean water and sanitation, while Goal 7 seeks to promote affordable and clean energy. Goal 8 aims to create decent work and foster economic growth, while Goal 9 focuses on innovation, infrastructure development, and sustainable industrialization. Goal 10 seeks to reduce inequalities within societies, and Goal 17 emphasizes the importance of partnerships to achieve these goals. Together, these interconnected SDGs define the essential requirements for creating a prosperous and sustainable future for humanity.

Digital technologies can play a critical role in achieving the SDGs by enabling the creation, use, communication, and storage of electronic information for organizational purposes. Digital resilience can be seen as the technology that helps achieve these goals. Sustainable economic growth that considers and integrates the 2030 Agenda for Sustainable Development [16] is the goal of the endeavor known as “a digital sustainable future,” which entails developing and deploying innovative technology. The value of cutting-edge digital inventions like Artificial Intelligence (AI) and Machine Learning (ML) has increased exponentially and is expected to contribute approximately 14 percent to the world's Gross Domestic Product (GDP) by 2030. In order to create a sustainable planet that provides resources while preserving the environment and the health of its inhabitants, it is important to explore how technology can pave the way for eco-friendly development. This involves identifying which Sustainable Development Goals (SDGs) can be effectively addressed by each industry.

In this article, we explore the pivotal role of technology in advancing Sustainable Development Goals (SDGs) across various sectors, including the food-water-energy nexus, businesses, public health, and global warming mitigation, and biodiversity conservation. For each significant challenge, we delve into how digitalization can facilitate the transition towards a society that is both environmentally sustainable and socially equitable. Specifically, we focus on how digitalization methods can directly benefit specific industries in achieving the aforementioned SDGs.

However, it is important to note the intricate interconnectedness among these topics, making them difficult to segregate. For instance, the health of individuals in interconnected cities is profoundly influenced by the interplay of their food, water, and energy systems. Thus, we examine how digitalization aids in preserving the food-water-energy nexus, optimizing production in factories, and enhancing public health and well-being. Additionally, we investigate how digitization can contribute to mitigating rising temperatures and safeguarding biodiversity [17].

Despite the challenges inherent in the evolution of sustainable practices, such as disjointed design methods and ecological technology substances, environmentally conscious cities are actively striving to become smarter and more sustainable in the era of big data [18]. Their success in meeting sustainability targets is closely tied to this endeavor, especially considering the unpredictable nature of climate change, economic volatility, epidemics, and population shifts in urban areas.

Cutting-edge Information and Communication Technology (ICT) plays a crucial role in overcoming these challenges, enabling a higher standard of living, sustained economic growth, and responsible management of natural resources. These technologies are essential for our understanding and development of environmentally friendly cities, which encompass a wide array of buildings, operations, goods, and services. The objectives of this study include following.

(1) To assess the existing integration of data-driven technologies and environmentally responsible concepts in smart city programmes.
(2) To determine any current deficiencies or inadequacies in the incorporation of these ideas within smart city frameworks.
(3) Investigate the capabilities of big data technologies in the surveillance, comprehension, and assessment of sustainable urban development.
(4) Evaluate the potential of smart city technology in promoting sustainability objectives in the face of rising urbanization.

To ensure sustainable growth in city management and operations, it is imperative to develop and implement innovative techniques and solutions. In response, ‘data-driven smart, feasible cities' have emerged, leveraging advancements in data and smart city technologies. These cities are better equipped to navigate the complexities associated with environmentally conscious urban areas and can effectively measure, evaluate, and improve sustainability-related outcomes.

Moving forward, it is crucial to dispel the notion that environmentally friendly cities and advanced technology are mutually exclusive. The multifaceted challenges facing environmentally conscious cities across various domains necessitate a comprehensive approach to urban development. Adopting a holistic perspective, rather than an ‘either/or’ mentality, is paramount for advancing sustainability in urban areas. The contributions of this study are as under.

(1) The paper evaluates the present condition of research in data-driven, environmentally concerned smart cities. It assesses the extent to which these cities are successfully integrating data-driven methods to improve sustainability.
(2) The paper addresses potential deficiencies in the realm of data-driven sustainable cities. Accurate identification is essential for guiding future research endeavours and guaranteeing complete strategies for urban sustainability.
(3) This study highlights the significance of using data-driven approaches to build strategic policies and operational procedures for managing sustainable cities. This indicates real ramifications for legislators and urban planners when enacting sustainable measures.
(4) The study highlights the importance of using data-driven advancements to ensure that environmentally conscious cities continue to contribute to sustainability goals, even in the face of rapid urbanization. It offers a roadmap for ongoing efforts in sustainable urban development.

The remaining paper is organized as follows: Section 2 presents the literature review, Section 3 outlines the methodology, Section 4 describes the results and discussion, and Section 5 presents conclusion and policy implications.

## 2 Review of literature

**Fig. 1:**
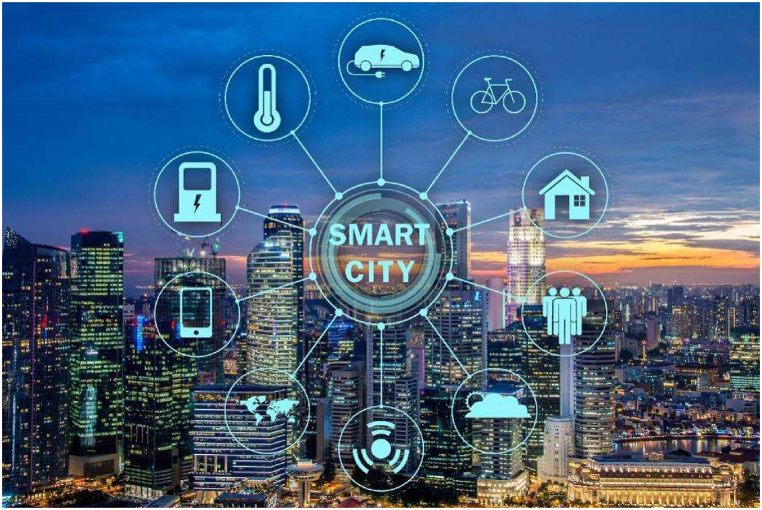
Various components and connections in a smart city [21].

The utilization of big data and technological advancements holds immense importance in monitoring and developing smart cities. Big data technology facilitates real-time data monitoring and analysis, leading to the optimization of city services, the promotion of sustainability, and the enhancement of public safety [19]. Moreover, it fosters the evolution of existing and the creation of novel business models, products, and services, thereby contributing to broader socio-economic development [20]. Fig. 1 indicates various components and connections in a smart city.

https://government.economictimes.indiatimes.com/news/smart-infra/tamil-nadu-govt-readies-warangal-master-plan/74587854.

**Fig. 2:**
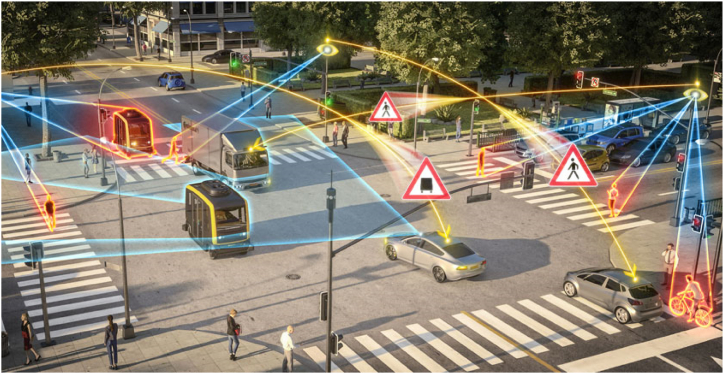
Concept of transport connectivity in a smart city [24].

The effective management of smart cities heavily relies on the efficient processing of large and diverse datasets, particularly in transportation. Through big data analytics and UAV-related technologies, costs and resource consumption can be minimized, route planning can be optimized, and emissions can be reduced [22]. By incorporating big data technology into smart city planning, construction processes can be expedited and improved [23]. In summary, big data and technological advancements play a pivotal role in enhancing the efficiency, sustainability, and overall quality of life in smart cities. Fig. 2 indicates the concept of transport connectivity is a smart city.

https://www.thenationalnews.com/lifestyle/motoring/driving-ambitions-what-our-roads-will-look-like-in-2050-1.901344.

Sustainable land management through the use of remote sensing and geographic information systems (GIS) offers various opportunities for crop species identification, sustainable farm and landscape-level management of agricultural systems, and policy development [25]. Integrating these cutting-edge digital technologies can improve several areas, including agricultural outputs, conservation of resources, both above and below-ground natural ecosystems, gender parity, and farmers' autonomy. Multiple instances demonstrate how integrating RS-GIS, Fuzzy-logic, and multi-criterion examination using the analytical hierarchy procedure can lead to better decisions regarding land utilization, crop diversity, and scheduling, monitoring, and overall agricultural ecosystem health, as outlined in Table 1. Soil survey data may be combined with other relevant datasets more quickly and efficiently by using these integrating methods, allowing for more accurate land-use adaptability evaluation [26]. Application of these methods is beneficial for highly efficient and stable systems of farming in the long run, according to studies. Better agricultural systems in producing regions more resilient to climate change may be achieved via the astute use of GIS-based technologies. In China's citrus fruit groves, for example, GIS-remote recognizing instruments have been used to forecast the appropriate area for citrus showing and their sustainable handling from growing to end-users through digital imagery and demonstrating of area elevation, land-use, various kinds of soil, climate variables, and higher elevations [27].The RS data is crucial in understanding the spatial and temporal features of the land, such as the effect of the environment on crop development. Sustainable potato cultivation could be enhanced through the use of GIS-RS technology and models, as demonstrated by Ref. [28]. They emphasized the importance of high-tech, multidimensional imagery in remote sensing (RS) as a tool for monitoring and assessing plant interactions, vegetation health, and predicting agricultural yields under different management practices. Their research highlighted several relevant parameters, including the Normalized Difference Vegetation Index (NDVI), Spectral Reflectance, percentage of Photosynthetically Active Radiation (PAR) absorption, LandSAT Thematic Mapper data, and Environmental Policy Incorporated Climate change models. As a result, the agricultural community could make more informed choices about the product quantity exported and imported within the area. The federal government benefits from GIS-RS-based technology since it can give the most important projects to assist farmers with accurate maps, crop information, field estimates, and soil features. These systems also give more accurate data on climate factors, which may be used in many breeding strategies to create heat- and drought-resistant crop varieties [29]. The vulnerability to severe subsidence of soil in Damascus with soil destruction of more than 109 tons was also explained by a map for soil deterioration generated by a GIS-RS that included Revised International Soil Loss Coefficient simulations [30]. Mulching, hail protection, crop insurance, shade nets, and irrigation assistance are among the many uses for the approximately 104–627 kg/ha of plastic materials and synthetic polymer substances currently utilized in agriculture. Continuous elimination of these macromolecules is essential for the long-term health of property, agricultural products, and soil biodiversity. Furthermore, immediately updatable GIS-based records and maps have been utilized to estimate the volume of waste material in farms, mark out the gathering area, and create an administrative and monitoring framework for the process of collecting and transporting recyclables to facilities for recycling. Those nations with a scarce or severely restricted water supply and dry and semi-arid regions must rely heavily on precision irrigation, which is where geographic information science and systems for decision-making come in. This method, however, requires merging extensive sets of exact and very reliable information on land features and water supplies. In underdeveloped nations, an application might be arduous due to factors including the high operation price and the sensors' certification. Open-source geographic frameworks like QGIS and R make it possible to use these technologies in economically disadvantaged systems even though they remain a barrier for many nations. Active participation from stakeholders is essential to further emphasize the availability and viability of such Geographic Information Systems (GIS) applications to the rest of the farming population and supply chain participants. Now more than ever, people have access to a wealth of knowledge at their fingertips because of the proliferation of mobile and desktop apps. There are several chances to dramatically enhance the sustainability on all levels of food manufacturing structures, and academic treatments may further promote the incorporation of developing digital technology inside the farming process.

**Table 1:** Densification characteristics.

Urbanformfeatures	Spatialfeatures	Socialfunctions
• thick urban areas	• Various Land Uses	• Justice in society
• Reduced reliance on private automobiles	• The life's Variety	• Independence in all facets of life
• Cut away from the rest of the neighborhood	• Absolute id	• Governmental autonomy

**Fig. 3:**
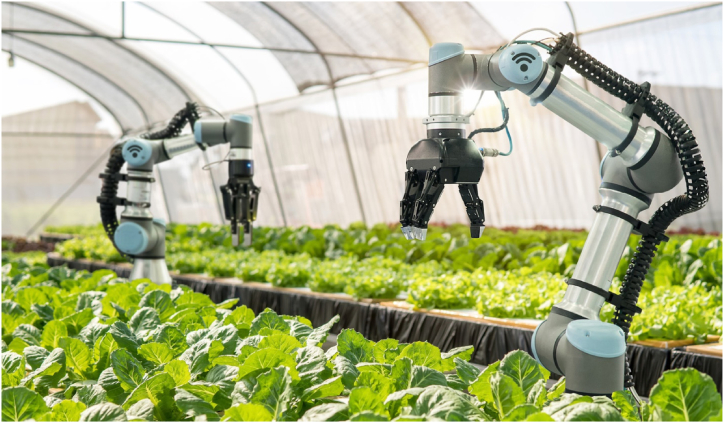
Concept of smart farming in context of smart cities [35].

Significant advancements are currently being made in the field of agriculture, thanks to ICT and digitalization. These technologies are driving innovation and transformation across the industry. Table 1 provides a summary of various ways in which agriculture partners are leveraging mobile application software to enhance resource utilization, reduce production costs, increase yields, and maximize profits [31]. Farmers, scientists, and technicians now have access to mobile-based apps that help them learn how to implement climate-smart farming methods. By having computerized accessibility built in, it aids in making choices across manufacturing and the rest of the supply chain. Real-time intelligence information compiled by the Global System for Mobile Computing Organization indicates that over 5.2 billion individuals (about 68 % of the world's inhabitants) utilize mobile phones or tablets. More than 3 billion mobile cellphone owners worldwide are expected to expand by several hundred million over the next few decades. Smartphones and apps efficiently transmit agriculture knowledge to farmers [32]. However, not all farmers see increased production and income after using ICT. Thus, many farmers continue to operate in the “dark” due to lack of modern technological access, particularly in rural areas in developing nations notwithstanding the favorable effects of mobile apps on enhancing the agriculture of smallholders [33]. Lack of communication, missing digital capacity, poor functionality of apps and websites, and digital literacy deficiencies are particularly significant barriers to technological advancement within the agricultural sector (i.e., the use of mobile phones/Apps and ICT), particularly among small-scale agricultural producers in rural surroundings. If these problems aren't fixed, rural communities will enter a new age of technological deprivation. As the new ICT cutting-edge programs have an opportunity to avert the looming digital destitution among small-scale agricultural producers recognizing the regional context of communication, user abilities, coordination with local languages, and socioeconomic status experiences should be prioritized [34]. Fig. 3 represents concept of smart farming in context of smart cities.

(https://www.app.com.pk/global/smart-agriculture-forum-in-beijing-attracts-pakistani-experts-entrepreneurs/)

The effect of climate change is a danger to the increasing productivity of farming, food availability, and livelihoods of multitudes of people worldwide. The release of greenhouse gases (GHGs) production and warming temperatures are both exacerbated by the agricultural sector [36]. argue that climate-smart farming methods based on technological advancements can solve current issues since they incorporate the advantages of environmentally friendly manufacturing, weatherproofing, and lower GHG emissions. Considering the many advantages of CSA technology, only a small percentage of agricultural enterprises have adopted them thus far. Implementing CSA technology is influenced by several factors, including farmers' economic circumstances, the biophysical conditions of a given site, and the features of contemporary technology. The food system requires a significant digital overhaul to deal with environmental degradation and food insecurity. From the world's sustainable development viewpoint, more environmentally conscious agriculture might be driven by the broad acceptance of APP-based farming services with geographically specific CSA methods.

### 2.1 Intelligent machines, robots, and agricultural precision

**Fig. 4:**
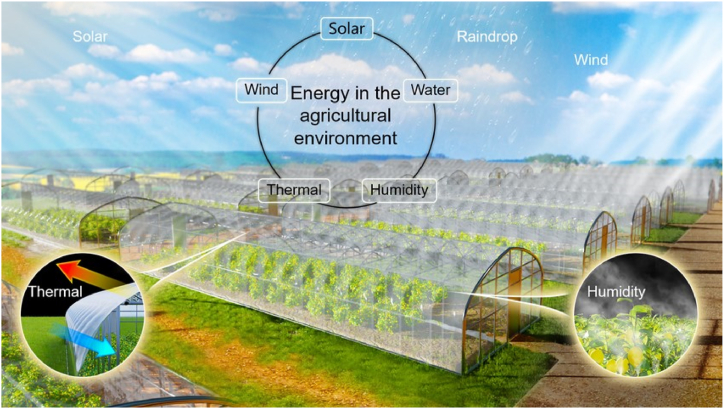
Concept of smart farming with renewable energy [38].

Instead of applying a single agricultural technique uniformly, precision gardening implements it at a precise location and time. Precision farming is defined by its core characteristics, which include lower ecological hazards, increased crop output (decreased world hunger), and financial benefits (decreased destitution) to communities that grow crops. Precision cultivation is a responsible kind of farming that uses cutting-edge technologies like robots, AI, and advanced intelligence to adapt to changing environmental conditions. Many issues plaguing agricultural systems and the agricultural sector can be alleviated, and the progress toward agricultural precision would be bolstered if modern unmanned aerial vehicles (UAVs) or drones could generate hyper-class remote-sensing dynamical and spectrum recordings. Utilizing various sensors on UAVs, real-time predictions can be made for a range of factors affecting agricultural production, including drought, soil nutrient levels, plant development, yield, disease outbreaks, insecticide and fungicide application, pest infestations, temperature variations, soil type, moisture content, and nutrient applications. Additionally, thermal imagery combined with hyperspectral data and entity-based image processing techniques allows for improved visualization of moisture stress, organism presence (weeds, beneficial insects, etc.), nutritional status, and potential yield outcomes, further enhancing UAV-based modeling capabilities. The effectiveness of accurate farming has also been enhanced by artificially intelligent technology. For instance, using cutting-edge AI examples, advanced reinforcement learning, data, and cloud-based computing might help farmers save money and the environment without sacrificing yield or quality [37]. In order to improve advising and information accessibility to the agricultural society as a whole, national governing authorities have also adopted the innovations of these interconnected technologies Expert platforms for cotton cultivation were created using intelligent machines in other studies; they include COMAX (Cotton Management eXpert) and COTFLEX. These technologies help farmers make informed decisions. Fig. 4 represents the concept of smart farming with renewable energy.

Triboelectric nanogenerators for smart agriculture.

Soybean crop insect assaults and the application of pesticides may also be gleaned from the SOYGRO and PRITHVI expert systems [39]. Agriculture IoT and automated learning solutions become possible thanks to AI. With the advent of wireless communications, farmers can access more information and make more informed decisions about farm management. Accelerating growth in precision agriculture may also be attributed to improvements in sensor technology, control mechanisms, and computerized photographic equipment enabled by ever-improving machine intelligence. Seedbed grounding or earthing water supply, application, trimming, gathering, continuous surveillance, and modeling are just some of the areas where semi- and fully autonomous, unmanned aerial machines or grounded automation are being effectively employed to save resources, money, and the natural environment [40]. Data represented in many forms, such as Virtual Agriculture, Agricultural 5.0, and Intelligent Growing food, improves the preciseness of farming operations and facilitates crucial operational choices in precision agriculture. Examples of sensors and technologies contributing to feasible use for farming development and administration include the United States satellites, Landsat, and Europe's Sentinel 2 and 3 satellite systems. These India's IRS satellites are the RapidEye constellation, the GeoEye-1 system, the Worldview series, and Japan's IKONOS. Vinbot, VineRobot, VineScout, and GRAPE are more examples of robotic technologies that contain crop sensor gadgets that allow for multi-season ground-truth assessment of vineyards. Also, two exciting automation technologies are Nao Innovations and Rowboat Technology LLC. The former is linked to the mechanized removal of weeds, while the latter is used in duties such as selected fertilizer application, monitoring crop development, etc. Implementation, which includes operational preparation and documentation for agricultural management of systems may all benefit from data collected by FISs at the field level [41]. Sustainable agricultural manufacturing: the use of genetics, computational biology, and massive amounts of data.

While traditional genome research, biological information technology, and big data analysis primarily focused on understanding fundamental biological mechanisms, modern omics integrated with advanced computational approaches have significantly bolstered Agri-sciences. This integration has led to breakthroughs in improving crop production, nutrient quality, stress adaptation, and resilience against climate change impacts. Forecasts predict that a 1-degree increase in global temperature could reduce corn yield by 7.4 %, grain yield by 6 %, rice yield by 3 %, and soybean yield by 3.1 %. Cultivating resilient, productive, and climate-resilient crops necessitates a deep understanding of crop genetics. Recent progress has seen draft genomes being sequenced for various grain crops (Oryza sativa, Setaria italic, Sorghum bicolor, Triticum aestivum, Zea mays) and legumes (Cicer arietinum, Cajanus cajan, Glycine max, Phaseolus vulgaris, Vigna radiata). Similarly, research on seed oils includes Brassica napus, Camelina sativa, and Elaeis guineensis. This wealth of genomic data, along with information from proteomics and metabolomics, is analyzed and managed through cutting-edge bioinformatics platforms and applications powered by computer and cloud technologies. These bioinformatics tools help us decipher the vast amounts of digital information generated in the agricultural sciences. Examples like the Rice Genome Annotation Project showcase dedicated databases built to safeguard food and nutritional security. By harnessing genomics, bioinformatics, and big data, we can improve crucial agricultural traits in crops, including: Tolerance to abiotic stresses such as drought or extreme temperatures, resistance to insects and diseases, herbicide tolerance, higher grain yield, plant height and architecture, and enhanced nutrient content.

These improvements pave the way for more sustainable and efficient food production, contributing to global food security and resilience. To enhance climate-resilient, high-yielding, nutrients crop cultivars [42], propose the 5Gs crop breeding approaches of crop-specific gene assembly, the genome and agronomic trait-based assessment of genetic material, recognizing genes combined with functional commentary, genomics development, and genetic modification. Furthermore, CRISPR-Cas9 aids in developing disease, drought, and herbicide-resistant crops with enhanced plant resistance against the virus, bioactive chemical synthesis, crop yield, and nutrient content thanks to pinpoint editing of genome technology. CRISPR-Cas9 is a genetic modification method that can benefit crops like wheat, maize, rice, tomato, and sweet orange. It is accomplished with the use of numerous targeting sgRNAs. Moreover, the combination of these technologies can help fight infections caused by Bean yellow dwarf virus (BeYDV), potato yellow curling of leaves virus (TYLCV), and Beetroot Severe Curly Top geminivirus (BSCTV). This technology has shown promise in modifying the genome of the virus. Additionally, CRISPR-Cas9 can be used to induce mutations in specific genes such as the TaMLO-A1 and TaMLO-B1 genes of wheat, the OsERF922 gene of rice, and the SIDMR6-1 chromosome of tomato. By doing so, it can increase the plant's susceptibility to powdery mildewing, rice blast, and the bacterium spp. Similarly, the ARGOS8 gene locus in maize has been edited using the CRISPER-based genome-modifying technique to increase grain production despite drought. Additionally, maize with a change in the acetolactate synthetase (ALS)1 or ALS2 chromosome has developed sensitivity to the pesticide chlorsulfuron [43].

### 2.2 Review of the state of art in methodology art

Within the realm of literature reviews, various methodologies offer distinct attributes and perspectives for analyzing subjects. These include systematic review, best-evidence synthesis, anecdotal review, meta-analysis, comprehensive review, umbrella review, and rapid assessment. Best-evidence synthesis, for instance, rigorously examines, assesses, and synthesizes research findings from different projects, reducing bias and ensuring the balanced incorporation of high-quality scientific evidence. This method is particularly suitable for focused themes [44], contrasting with the multifaceted nature of data-driven smart cities that encompass multiple issues and research strands.

In the context of this research, the literature review methodology employed is broad in scope and individualized, deviating from a predefined protocol. It adopts a narrative review approach, integrating diverse sources of research and incorporating reviewers' personal experiences, assumptions, and perspectives. Narrative reviews are well-suited for extensive subjects [45]. In addition to the narrative approach, the best-evidence synthesizing strategy is employed to analyze a wide range of evidence and consider contextual influences. This approach complements the narrative review method by gathering all relevant material on a study subject, identifying knowledge gaps, establishing an evidence base for effective guidelines, or informing policymakers and practitioners. It is preferred over a cinematic review, which focuses on the author's viewpoint, to provide rationale for study selection, therapy efficacy, and the relevance of selection criteria. By offering detailed information about original studies, audiences can draw independent conclusions, as highlighted by Ref. [46]. Overall, this research aims to demonstrate the value of integrating these two fundamental literature review approaches.

**Fig. 5:**
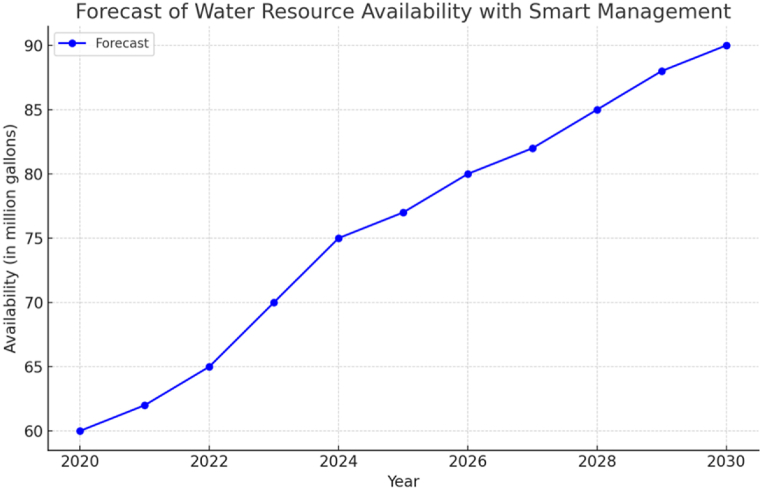
Forecast of water resource availability with smart management.

Regarding the aims of literature reviews, they may encompass methodological, theoretical, topical, and state-of-the-art objectives. This literature study primarily focuses on modern developments and topical objectives. The former addresses recent research, outlines future directions, and synthesizes contemporary thinking, providing fresh insights. The latter targets specific areas of scientific literature, aiming to identify deficiencies and pave the way for change. It offers an in-depth assessment of concepts through the examination of objectives, as illustrated in Fig. 5.

## 3 Methodology

### 3.1 Integrated and multidimensional viewpoints

Academic assessment stands as a cornerstone in scholarly activities across various fields and sectors of science. This literature review delves into the important topic of data-driven intelligent cities with a sustainable focus, examining, evaluating, generating, and analyzing a substantial body of studies. It revolves around the underlying models of urbanization and their integration to promote sustainability objectives. Drawing from diverse city-academic domains and practical findings from numerous case studies, the review aims to provide a comprehensive understanding of the subject matter.

The collaboration between disciplines and multidisciplinary thinking has become increasingly prominent, resulting in a surge of academic publications. The study of data-driven environmentally conscious cities inherently demands a multidisciplinary and interdisciplinary approach. It recognizes that advancements in understanding require exploration of multidimensional topics, which can only be addressed through collaboration and transdisciplinary perspectives. As such, research within this domain is characterized by its complexity and constant evolution, necessitating contributions from various professionals.

In academia, multidisciplinary and interdisciplinary research methods are employed to analyze this literature. Multidisciplinary approaches emphasize the integration of disciplines to generate new insights and knowledge beyond traditional boundaries. They leverage ideas and methodologies from diverse fields to enrich analyses and transcend disciplinary constraints. Conversely, interdisciplinary methods focus on the synthesis of different fields, combining ideas and techniques to yield outcomes that surpass the sum of their parts. Transdisciplinary approaches bridge the gap between disciplines, integrating specific fields of study to explore the intersections and generate holistic insights.

### 3.2 Combinatorial search technique and academic sources

A literature search involves searching through academic literature resources to obtain research articles related to the examined issue. A search approach was applied, including numerous computerized search databases, including Scopus and ScienceDirect. This was SpringerLink, Sage Journals, and the search engine Google Scholar. The most significant donations come from prominent journal papers. The hierarchical search technique involves: • Searching approved high academic literature repositories.

• Searching evidence-based publications for reviewing articles; and
• Routine inquiries and other searching providers.

Additionally, the gathering procedure is based on Scott's (1990) four criteria for judging the quality of the desired content, namely.

• Authenticity: the information acquired is genuine and of undeniable origin.
• Credibility: the information acquired is free from inaccuracy and manipulation.
• Representation: the evidence collected is conventional.
• Meaning: the information acquired is straightforward and intelligible.

### 3.3 Selecting criteria: inclusion as well as exclusion

**Fig. 6:**
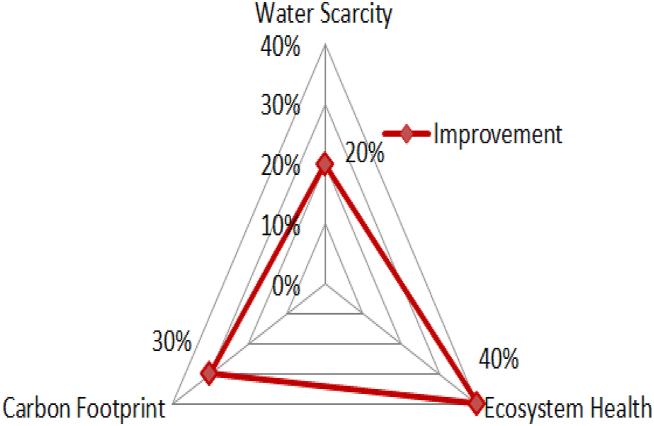
Contribution to geo-sustainability indicators.

In order to understand the dynamic field of data-driven smart cities with a focus on environmental awareness, we used search method mentioned in section 3.2 to find relevant research studies. This strategy is focused on multiple study streams within the subject, specifically addressing six crucial concerns. At first, the available information was evaluated in accordance with the research difficulties. Therefore, it was considered appropriate to limit the search and concentrate on particular literature sources. Nevertheless, it was observed that multiple sources of information seemed relevant to the main issue. Hence, in order for a written piece to be considered valuable, it must align with one of the theoretical themes or sets of issues specified in this publication. The themes closely correspond to the titles of the sections and subsections of this study. Fig. 6 illustrates the contribution to geo-sustainability indicators.

**Fig. 7:**
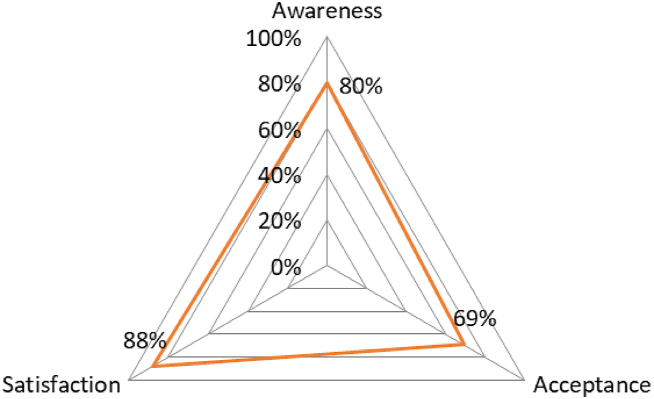
Public perception and acceptance rates.

Our objective was to gather an extensive set of relevant research. The materials were assessed according to their efficacy in tackling the issues, with different degrees of significance ascribed to each, depending on the chosen conceptual themes and subject subcategories. The latter sought to highlight crucial elements of data-driven, sustainable cities. On the other hand, materials that were not relevant to the current issues were not included. Nevertheless, certain sources provide insight into the planning and development ideas that endorse environmentally conscious cities as a component of a comprehensive approach. The summaries underwent meticulous evaluation to ascertain their relevance, and strict criteria for inclusion and exclusion were regularly employed. Reviewing abstracts again assisted in resolving issues regarding what should be included. Fig. 7 displays the level of public perception and acceptability.

**Fig. 8:**
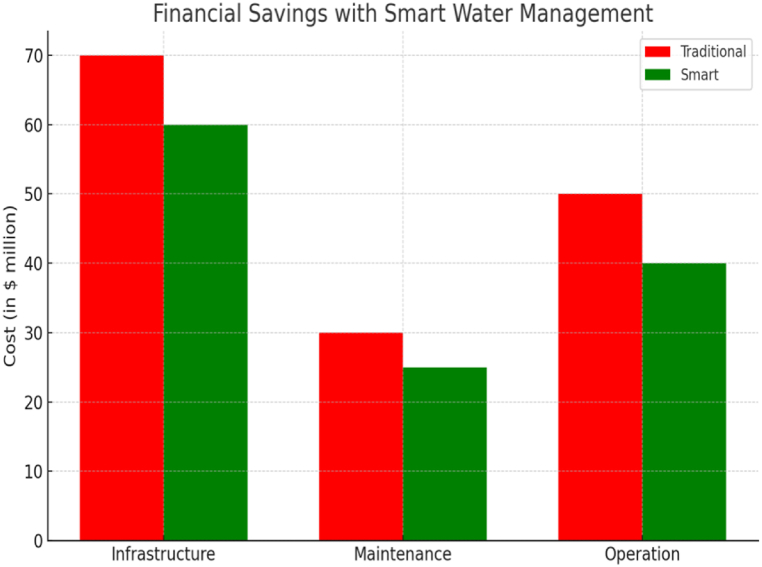
Financial savings with smart water management.

A variety of terms related to urban areas preparation, administration, and cognitive ability. These terms were then used to search the paper titles, abstracts, and keywords to generate preliminary hypotheses. Backward literature examination (using previous authors and previous references) in addition to forward research search (using current authors and current connections) were utilized to supplement the search term technique. Fig. 8 represents financial savings with smart water management.

### 3.4 Reasons for existence and methods of organization

There are several reasons why a literature review could be conducted. This varies on whether it is prompted by, or an essential part of a scientific study and thus its breadth or area of concentration. This indicates the amount to which the investigation field will be studied in the investigation, which effectively implies outlining whatever the study encompasses and what it concentrates on. The current literature review is for publishing and thus was performed with the objective of:

Data-driven smart ecological cities: a combined form of urbanism, described and discussed as a theoretical framework.

Data-driven individual smart unsustainable cities: analyzing, evaluating, and synthesizing the current understanding provide a critical evaluation of the prior study by emphasizing its merits, shortcomings, and inconsistencies; analyze the stated benefits and drawbacks of technologies based on information and environment; determine the possibilities, advantages, and future outlook for the sustainability of cities brought forth by data-driven technology; and compare, connect, and leverage the relevant research findings addressing the many strands of the issue to establish substantial connections among them.

## 4 Results and discussion

The discourse surrounding sustainable cities has undoubtedly expanded significantly and garnered considerable attention. However, despite this growing conversation, concrete definitions of what constitutes a sustainable city remain elusive and subject to ongoing debate. While the concept of sustainable cities enjoys widespread endorsement as a policy objective, the specific strategies and actions required to realize this vision are still in need of clarification.

**Fig. 9:**
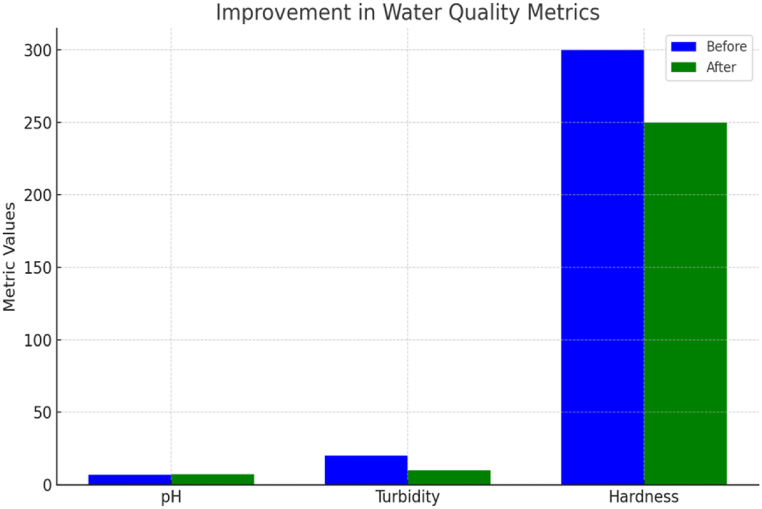
Improvement in water quality metrics.

Fig. 9 highlights the complexity and nuances involved in defining and understanding sustainable cities. It emphasizes the multifaceted nature of sustainability, encompassing various dimensions such as environmental, social, economic, and cultural aspects. Moreover, it underscores the interconnectedness of these dimensions and the necessity for holistic approaches to urban development.

In essence, while there is broad agreement on the importance of building sustainable cities, the precise pathways to achieving this goal are complex and multifaceted. Addressing this complexity necessitates ongoing dialogue, research, and collaboration among policymakers, urban planners, academics, and community stakeholders. Together, they must develop comprehensive strategies that can effectively balance environmental protection, social equity, and economic prosperity in urban environments.

Given the intricate and abstract nature of environmentally conscious cities, the term is interpreted differently across various fields that study urban environments, such as engineering, sociology, and computer science. Consequently, there exist numerous conceptualizations of what constitutes an environmentally friendly city.

In essence, an environmentally friendly city embodies a systematic approach to urban development and construction that prioritizes sustainability from the outset. It serves as a framework for realizing the long-term objectives of sustainable urban development, functioning as a strategic planning tool. Central to this concept is the need to strike a delicate balance among the monetary, ecological, and human dimensions of sustainability.

**Fig. 10:**
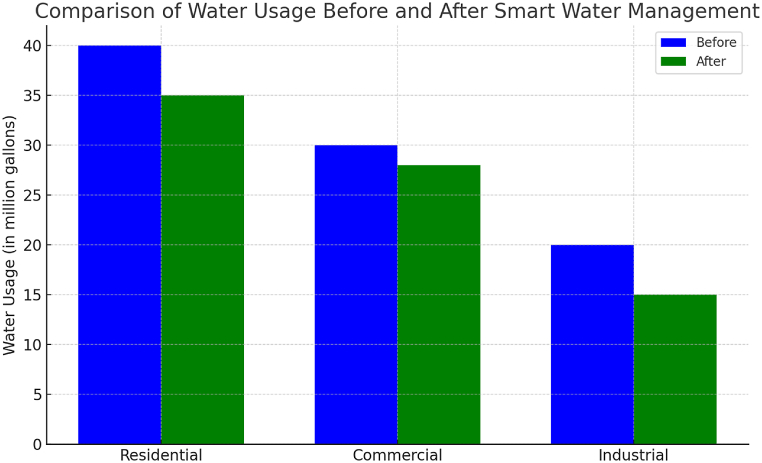
Comparison of water usage before and after smart water management.

Fig. 10 illustrates the necessity of finding a harmonious equilibrium among these pillars of sustainability within the context of environmentally friendly cities. This balance is crucial for ensuring that urban development initiatives not only mitigate environmental degradation but also promote social equity and economic prosperity. By integrating environmental considerations into urban planning processes, environmentally friendly cities strive to create livable, resilient, and inclusive urban environments for present and future generations.

The goals of an environmentally conscious town, as a comprehensive method of change, encompass various aspects aimed at promoting sustainability and reducing environmental impact. These goals include maximizing resource efficiency, minimizing waste generation, promoting renewable energy utilization, achieving carbon independence, reducing pollutants, establishing environmentally friendly transportation systems, emphasizing compact and adaptable urban design, preserving biodiversity and open spaces, and fostering accessibility and community-oriented living conditions. In essence, a sustainable town aims to meet the needs of its current residents without compromising the ability of future generations to meet their own needs.

Models for environmentally friendly urban forms can be derived from various concepts, including new urbanism, ecocities, urban containment, environmental urbanization (which combines landscape architecture and urban ecology), and compact cities, among others. However, smaller towns and ecocities are often highlighted as the most frequently used and promoted examples of sustainable urban forms, representing core concepts of ecologically conscious urbanism. Compact towns and eco-cities, in particular, are widely accepted and supported concepts for achieving environmentally friendly urban development.

**Fig. 11:**
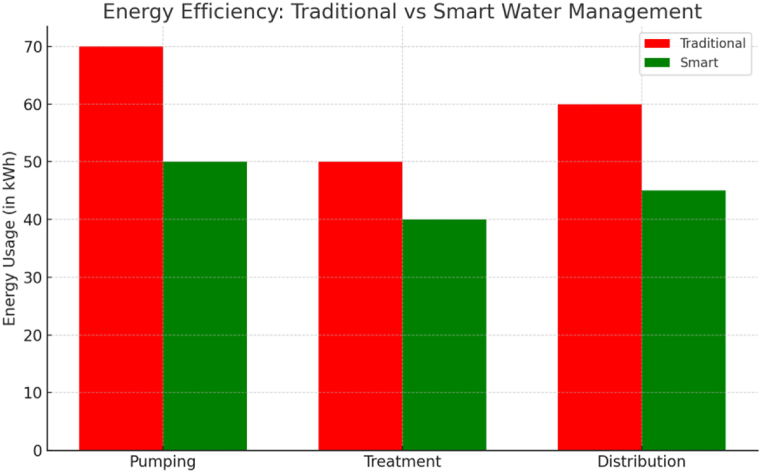
Energy efficiency: traditional vs smart water management.

According to Ref. [47], environmentally friendly urban forms are characterized by compression in various forms, mixed land uses, interconnected street layouts, efficient transportation systems, environmental protection measures, and effective urban governance. This definition suggests a hybrid form that combines elements of compacted and eco-city layouts, highlighting the importance of integrating sustainable design principles and governance structures to create environmentally conscious towns. Fig. 11 provides a visual representation of these concepts and their interconnectedness.

In contrast to the compressing city, where engineering is at the center of compacting methods, management tends to take center stage in the eco-city. That indicates that eco-city is less concerned with the spatial arrangement of the city's defining physical features than it is with the planning and managing of the urban environment. These two sustainability urban designs have several shared ideas, conceptions, and ideals.

### 4.1 Dense urban areas

The term “compact city” lacks a precise definition in the academic literature despite its widespread recognition. According to Ref. [48], a “compact city” generally refers to a high-density, mixed-use urban development characterized by an effective public transportation infrastructure and features designed to encourage walking and cycling. On the other hand, perspectives such as those presented by Ref. [49] describe the urban core of a compact city as having higher density and integrated land use without sprawl.

Densification characteristics of compact cities can be understood through three main factors: urban form, the environment, and social functions, as outlined in Table 1. Urban form features include thick urban areas, reduced reliance on private automobiles, and a separation from surrounding neighborhoods. Spatial features entail various land uses and a diverse mix of life activities, while social functions encompass principles of justice in society, independence in various aspects of life, and governmental autonomy.

For a more comprehensive examination of the fundamental challenges associated with compact cities, interested readers are encouraged. Overall, the concept of a compact city underscores the importance of dense, mixed-use urban development that promotes sustainability, walkability, and social cohesion.

In contrast to the social and financial aspects of resilience eco-cities prioritize environmental sustainability concerning the surrounding environment and ecosystems. While the natural environment has always been a consideration in urban development, eco-cities elevate this focus to the forefront of urban planning and design. A comprehensive literature analysis is conducted to identify three broad categories of eco-city models as outlined in Table 2.

**Table 2:** Three types of eco-city models.

Type 1	Type 2	Type 3
• Eco-community	• Eco-City	• City of Perpetual Cooperation
• The Solar City	• Eco-District	• Clean Air Municipality
The Solar Town	• Ecological Metropolis	• Waste-Free Urban Area
• Cohousing	• Verdant Town	• No-Carbon Town
• Habitat Preservation	• Garden Town	• Carbon Neutral Neighborhood
	• Ecologically Sound Community	• Clean Air Community
	• Responsible Neighborhoods	• The Pervasive Eco-City
	• Metropolitan Sustainability	• Eco-City Technology
	• Robots with a pulse	• Intelligent Ecocity Powered by Information

Type 1 eco-cities prioritize the use of passive solar architecture to reduce energy consumption and minimize environmental impact. Type 2 eco-cities combine passive solar architecture with sustainable development practices, integrating environmentally friendly design principles into urban planning. Type 3 eco-cities emphasize innovative energy and environmental technologies, aiming for high levels of sustainability and efficiency. This category is associated with the concept of intelligent eco-cities, which align with the current movement toward environmentally conscious and technologically advanced urban planning for future generations.

Table 2 provides examples of different types of eco-city models, each with its own set of characteristics and goals. These models range from eco-communities and solar cities to clean air municipalities and waste-free urban areas. Each model represents a unique approach to creating sustainable, environmentally friendly urban environments that prioritize ecological preservation and resource efficiency.

The study distinguishes itself from previous research in the subject of sustainable urban development by its emphasis on data-driven methods, thorough evaluation of smart cities, identification of deficiencies and obstacles, dedication to evidence-based research, and offering of practical recommendations. Moreover, it distinguishes itself by not only highlighting successful implementations and best practices, but also by identifying potential gaps and issues in the sector. By identifying specific locations that require additional investigation or intervention, it provides significant information for progressing sustainable urban development initiatives. This study places particular emphasis on data-driven approaches, specifically big data applications, in promoting sustainability in the context of urban development. While other studies investigate different aspects of sustainable urban development, this study explicitly focuses on the importance of data-driven approaches. This study explores the ways in which these technologies can be used to tackle intricate urban problems, distinguishing it from other studies that may concentrate on alternative approaches or solutions. Moreover, it study provides a comprehensive evaluation of data-driven, environmentally conscious smart cities, going beyond superficial analysis often seen in other studies. The evaluation assesses the degree to which these towns are incorporating data-driven methods to enhance sustainability, offering a thorough comprehension of the present situation. This study not only makes scholarly contributions but also offers practical guidance for policymakers and urban planners. By emphasizing the policy implications of its results and providing recommendations for strategic development policies and operational management procedures, it closes the divide between research and practical implementation.

## 5 Conclusion and policy implication

In the contemporary era, cities embracing information and environmentally conscious principles are evolving into the epicenters of population growth and knowledge expansion at an accelerating pace. The potential of advanced Information and Communication Technology (ICT) for fostering a sustainable tomorrow is most evident in these cities. The convergence of efforts to integrate eco-friendly neighborhoods with intelligent urban systems has been catalyzed by numerous advancements. Amidst the era of big data, there is a prevailing belief in the transformative power of innovative urban strategies, technological progress, and institutional reforms in shaping intelligent and sustainable urban processes. Our study surveyed recent research on data-driven, intelligent, and environmentally conscious cities, aiming to provide a comprehensive examination and synthesis of existing literature. We elucidated established and emerging frameworks for sustainable development and intelligent cities, highlighting their interconnections and synergies.

The focus primarily rested on data-driven cities prioritizing environmental consciousness, comparing and contrasting compact towns and eco-cities as primary models for environmentally sound urbanism. We delved into their strengths, weaknesses, and opportunities for implementing innovative solutions to enhance societal sustainability. Subsequent sections explored the complexities and challenges of fostering environmentally friendly urban environments, considering social factors influencing informed decision-making in sustainable urban development. The study delineated technological alternatives for operations management and future planning, aiming to sustain the achievements of environmentally conscious cities in their pursuit of sustainability goals. Moreover, it critically evaluated potential risks and negative repercussions associated with data-driven urban planning, emphasizing the need to address techno centrism and bureaucratic tendencies. This study underscores the transformative potential of big data technology in revolutionizing sustainable urban development, advocating for development planning and operational strategies driven by data-driven innovations.

### 5.1 Policy implications

Policymakers should prioritize investments in data infrastructure to facilitate the gathering, analysis, and utilization of big data for sustainable urban development. This will entail efforts to strengthen data gathering procedures, facilitate data exchange among stakeholders, and allocate resources to technologies for data processing and analysis. Policies should promote the incorporation of data-driven methodologies into urban planning processes. This entails integrating data analytics tools and methodologies into decision-making processes, ensuring that urban development plans are guided by data-driven insights on sustainability. Policymakers should allocate resources to capacity building and training initiatives in order to provide relevant stakeholders with the necessary skills and knowledge to effectively utilize big data for sustainable urban development. One possible approach is to provide instruction in data analytics, data visualization, and other pertinent subjects to urban planners, policymakers, and other professionals. Moreover, due to the sensitive nature of the data used in smart city projects, legislators may need to create legislation and policies to protect data privacy and security. This may entail developing steps to protect data, establishing ownership rights over data, and ensuring adherence to applicable data privacy laws and regulations. Policies should give priority to include the community in the process of developing and implementing data-driven initiatives for sustainable urban development.

Future research endeavors should further explore the intricacies of sustainable, data-driven urbanism, addressing technological and policy challenges to advance environmental responsibility and foster harmonious integration of urban sustainability and technological progress.

## Ethics approval and consent to participate

Not applicable.

## Consent for publication

All of the authors consented to publish this manuscript.

## Data availability

All relevant data in form of figures and results have been included in this paper. Corresponding author (dr_lijian8218@163.com) may be contacted for any further query regarding data availability.

## CRediT authorship contribution statement

**Xiyin Ma:** Writing – review & editing, Methodology, Formal analysis. **Jian Li:** Writing – review & editing, Formal analysis, Data curation. **Zhiming Guo:** Writing – review & editing, Writing – original draft, Methodology, Formal analysis, Conceptualization. **Zhonglu Wan:** Writing – original draft.

## Declaration of competing interest

The authors declare that they have no known competing financial interests or personal relationships that could have appeared to influence the work reported in this paper.
